# Bias, Spin, and Misreporting: Time for Full Access to Trial Protocols and Results

**DOI:** 10.1371/journal.pmed.0050230

**Published:** 2008-11-25

**Authors:** An-Wen Chan

## Abstract

An-Wen Chan discusses the implications of a new study in*PLoS Medicine* that suggests that the randomized trial literature is skewed towards reporting favorable results.

Although randomized trials provide key guidance for how we practice medicine, trust in their published results has been eroded in recent years due to several high-profile cases of alleged data suppression, misrepresentation, and manipulation [1–5, 39]. While most publicized cases have involved pharmaceutical industry trials, accumulating empiric evidence has shown that selective reporting of results is a systemic problem afflicting all types of trials, including those with no commercial input [6]. These examples highlight the harmful potential impact of biased reporting on patient care, and the violation of ethical responsibilities of researchers and sponsors to disseminate results accurately and comprehensively.

Biased reporting arises when two main decisions are made based on the direction and statistical significance of the data—whether to publish the trial at all, and if so, which analyses and results to report in the publication. Strong evidence for the selective publication of positive trials has been available for decades [7,8]. More recent cohort studies have focused on the misreporting of trials within publications by comparing journal articles either with documents from regulatory agencies [9–12] or with trial protocols from research ethics committees [13–16], funding agencies [17], research groups [18,19], and journals [20]. These cohort studies identified major discrepancies—favorable results were often highlighted while unfavorable data were suppressed; definitions of primary outcomes were changed; and methods of statistical analysis were modified without explanation in the journal article.

**Linked Research Article**

This Perspective discusses the following new study published in *PLoS Medicine*:

Rising K, Bacchetti P, Bero L (2008) Reporting bias in drug trials submitted to the Food and Drug Administration: A review of publication and presentation. PLoS Med 5(11): e217. doi:10.1371/journal.pmed.0050217

Lisa Bero and colleagues review the publication status of all efficacy trials carried out in support of new drug approvals from 2001 and 2002, and find that a quarter of trials remain unpublished.

## New Evidence

In a new study published in *PLoS Medicine*, Lisa Bero and colleagues make an important contribution to the growing body of evidence that the randomized trial literature is skewed towards reporting favorable results [9]. The researchers identified trials from 33 new drug applications (NDAs) for new molecular entities approved by the United States Food and Drug Administration (FDA) in 2001–2002, and compared information from FDA reviews with journal articles. By including all NDAs from a variety of specialty fields, their findings have broad generalizability to pharmaceutical trials.

Overall, a substantial amount of primary outcome data submitted to the FDA was found to be missing from the literature. One quarter of trials in their sample were unpublished—predominantly those with unfavorable results. Not only were data suppressed for the unpublished trials, but an additional quarter of primary outcomes were omitted from journal articles of published trials. These findings are consistent with two recent reviews of FDA documents and journal articles [10,21], one of which was published in *PLoS Medicine* in September 2008 [21].

Bero and colleagues also identified important discrepancies between the primary outcomes, statistical analyses, and conclusions presented in NDAs versus those reported in journal articles. The vast majority of discrepancies favored the sponsor's new drug, suggesting biased reporting. While it is possible that the FDA requested modifications to the sponsor's analyses, these amendments should be mentioned in the FDA's statistical review; should not involve altering primary outcomes without explanation in the publication; and would not be expected to favor the sponsor's drug as often as was found in this study.

Biased reporting of results from NDA trials is particularly concerning because these journal articles are the only peer-reviewed source of information on recently approved drugs for health care providers, who will have had limited clinical experience with these new treatments. There are also substantial cost implications if the efficacy is overestimated and the drugs overused, as new molecular entities are among the most expensive pharmaceuticals on the market [22].

## The Need for Increased Transparency

Since the interests of patients are of utmost importance, it is difficult to justify why health care providers and policy makers should have access to only a biased subset of information that is substantially different from that which regulatory agencies have at their disposal. Bero and colleagues' study highlights the importance of public access to key documents that have traditionally been deemed confidential—regulatory agency submissions and trial protocols. Both types of documents have unique properties that complement each other.

Regulatory agency submissions represent the final description of how the trial was conducted and analyzed prior to journal publication. However, details from these submissions are not publicly available in most countries. Although summaries of FDA reviews are posted on the FDA Web site, their content and availability is variable, and sections are often redacted [9,21,23]. Furthermore, regulatory agency submissions are prepared by companies after data analysis and may themselves be subject to biased reporting. Finally, only devices, pharmaceuticals, and biological agents require regulatory approval in the United States and other countries, meaning that trials examining other types of interventions (e.g., surgery, education)—which constitute 20% of published randomized trials [24]—would be excluded from reviews of regulatory agency documents. Pharmaceutical trials conducted post-approval would also be missed.

On the other hand, protocols constitute the most comprehensive description of study design prior to trial inception. Their content therefore cannot be influenced by the study results. However, access to trial protocols is particularly difficult to obtain [25,26]. As with summaries of FDA reviews, their content is also highly variable and often lacks sufficient detail [13–18,20]. The SPIRIT initiative (Standard Protocol Items for Randomized Trials) aims to address these deficiencies by producing evidence-based recommendations for key information to include in a trial protocol [27].

## Time for Action

It is clear that the trial literature is biased, facilitated in part by limited oversight and difficulty in accessing detailed trial documents. Ongoing progress in trial registration and results disclosure represents a key initial step towards ensuring public access to basic information on trial methods and results [28–33]. Several journals have also acted by publishing protocols and requiring their submission with manuscripts [34–36].

However, much remains to be done—not only to establish reliable, comprehensive registration and results disclosure processes worldwide, but also to start heeding the calls for increased access to full protocols and regulatory agency submissions [14,23,33,37,38]. As shown by recent examples and studies highlighted above, misreporting of trials can be difficult to detect without access to detailed documents beyond what is currently available on registries and results databases. Only with full transparency can the validity of a randomized trial be judged.

The time has come to tackle the challenge of making key trial documents public. It has taken decades for trial registration and results disclosure to be implemented; hopefully, for the sake of patients, public access to full protocols and regulatory agency submissions will come much sooner.

## Abbreviations

FDA: Food and Drug Administration
NDA: new drug application

## Note Added in Proof

Reference 39 was added after this article was already in proof.
